# The Nanostructure of Myoendothelial Junctions Contributes to Signal Rectification between Endothelial and Vascular Smooth Muscle Cells

**DOI:** 10.1371/journal.pone.0033632

**Published:** 2012-04-16

**Authors:** Jens Christian Brasen, Jens Christian Brings Jacobsen, Niels-Henrik Holstein-Rathlou

**Affiliations:** Department of Biomedical Sciences, University of Copenhagen, Copenhagen, Denmark; Université Joseph Fourier, France

## Abstract

Micro-anatomical structures in tissues have potential physiological effects. In arteries and arterioles smooth muscle cells and endothelial cells are separated by the internal elastic lamina, but the two cell layers often make contact through micro protrusions called myoendothelial junctions. Cross talk between the two cell layers is important in regulating blood pressure and flow. We have used a spatiotemporal mathematical model to investigate how the myoendothelial junctions affect the information flow between the two cell layers. The geometry of the model mimics the structure of the two cell types and the myoendothelial junction. The model is implemented as a 2D axi-symmetrical model and solved using the finite element method. We have simulated diffusion of Ca^2+^ and IP_3_ between the two cell types and we show that the micro-anatomical structure of the myoendothelial junction in itself may rectify a signal between the two cell layers. The rectification is caused by the asymmetrical structure of the myoendothelial junction. Because the head of the myoendothelial junction is separated from the cell it is attached to by a narrow neck region, a signal generated in the neighboring cell can easily drive a concentration change in the head of the myoendothelial protrusion. Subsequently the signal can be amplified in the head, and activate the entire cell. In contrast, a signal in the cell from which the myoendothelial junction originates will be attenuated and delayed in the neck region as it travels into the head of the myoendothelial junction and the neighboring cell.

## Introduction

Information processing in tissues often relies on unidirectional flow of information. Such unidirectional flow is found in e.g. synapses of the nervous system [1]. Similar specialized anatomical structures that potentially enable signal rectification are also found in arteries and arterioles. Such vessels consist of a single layer of endothelial cells (ECs, see Table 1 for a full list of abbreviations), which lines the lumen, surrounded by one or more layers of smooth muscle cells (SMCs). The two cell types are separated by the internal elastic lamina [2], [3]. However, ECs and SMCs make occasional contacts through myoendothelial (i.e. muscle-endothelial) junctions (MEJs), which are mushroom shaped protrusions that project from one cell layer and traverse the internal elastic lamina to make contact with the other layer [2]–[5]. The MEJ can extend from either cell layer depending on the tissue and organism [2]–[4]. Gap junctions in the MEJ connect the cytoplasm of the two cells and are important in myoendothelial signal transduction [6], [7]. The gap junction itself is permeable to ions and small molecules (<1 kDa) including Ca^2+^ and IP_3_ [8]–[11].

**Table 1:** List of abbreviations.

Abbreviation	Name
EC	Endothelial cell
SMC	Smooth muscle cell
MEJ	Myoendothelial junction
ER	Endoplasmic reticulum
IP_3_	Inositol 1,4,5-trisphosphate
IP3R	Inositol 1,4,5-trisphosphate receptor (a Ca^2+^ channel)

The functional properties of the MEJ have been studied in mouse and rat mesenteric arteries where they extend from the EC layer to contact the SMC layer [6], [7]. A Ca^2+^ signal initiated in the SMC layer (e.g. by stimulation with phenylephrine) is apparently transmitted to the ECs by diffusion of IP_3_, whereas propagation of the signal in the opposite direction does not take place [12], . The directionality has been proposed to be due to rectification in heterodimeric gap junctions or to differences in distribution of IP_3_-receptor channels and other proteins [14]. However, the micro-anatomical structure of the MEJ has never been considered in this regard, and here we show that the structure of the MEJ alone can rectify a signal, which is mediated by a diffusible molecule between the two cell layers. The model shows that in an arteriole, the structure of the MEJ enables a diffusible molecule to propagate from the SMC into the MEJ from where it can spread into the body of the EC. In contrast, signaling in the opposite direction is less likely to happen due to the structure of the MEJ.

## Materials and Methods

### Description of the Models

**Figure 1:**
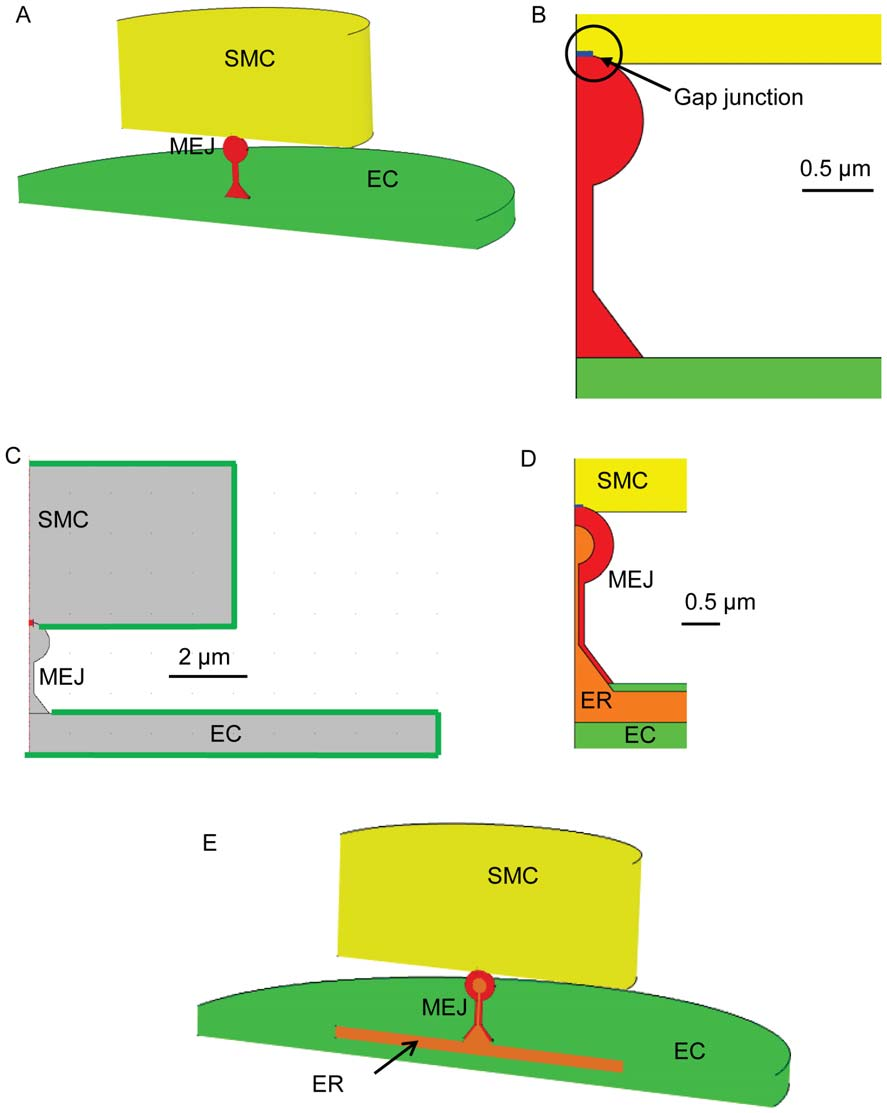
Description of the models. A, B) Model of the interaction between a smooth muscle cell (SMC) and an endothelial cell (EC) through the myoendothelial junction (MEJ). The SMC is shown in yellow, the EC in green and the MEJ in red. The SMC and MEJ are coupled in the area highlighted with the black circle at the blue edge. B,C) The endothelial cell (EC) has a radius of 10 µm and is 1 µm thick. The smooth muscle cell (SMC) has a radius of 5 µm and is 4 µm thick. The head of the MEJ is spherical with a radius of 0.5 µm that is located on top of a neck that is 1.525 µm long. Stimulation was modeled by increasing the concentration at the green boundaries in the respective cell. Scale bars indicate the respective dimensions. D,E) The model was extended to include endoplasmic reticulum (ER) in the EC. The ER had a radius of 6 µm, 0.5 µm thick and located 0.1 µm from the plasma membrane. The SMC is shown in yellow, the EC in green, the MEJ in red and the ER in orange.

To address the apparent asymmetry in myoendothelial signaling we constructed a spatiotemporal model of the EC-SMC contact (Fig. 1A–B). The structure is based on electron microscopic images of small rat and mouse mesenteric arteries [5], [15]. We assumed radial symmetry around the long axis of the MEJ (Fig. 1A) which enabled us to implement the model as a 2D axi-symmetrical model (Fig. 1B–C).

The model consisted of three main regions, the SMC, the EC and the MEJ that protrudes from the EC in the present model. The protrusion is in continuity with the EC and the SMC is connected with the MEJ through gap junctions, which in the model initially were assumed to have a lumped pore radius of 100 nm (Fig. 1B, marked with blue). Chemical species can diffuse between the cells through the gap junctions. In the model the head of the MEJ is spherical with a radius of 0.5 µm and located on top of a neck which is 1.525 µm long and has a radius of 0.125 µm. As shown in Fig. 1 the EC and SMC have different morphology. The EC has a radius of 10 µm and is 1 µm high whereas the SMC has a radius of 5 µm and is 4 µm high. Hence, we have for simplicity assumed that EC and SMC volumes are equal as also suggested in previous work on myoendothelial signaling [16].

Diffusion and chemical reactions were modeled using a diffusion-reaction equation,
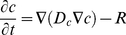
(1)


where *c* is the concentration of the diffusible species, *D_c_* is a diagonal matrix where the elements in the diagonal are the diffusion coefficients for *c* in the *x* and *z* directions, and *R* expresses chemical reactions and transport e.g. buffer reactions.

The model was solved numerically using the finite element method. The model was implemented in Comsol Multiphysics 4.1 (Comsol AB) [17] and was meshed with triangles using the built-in mesh function. Maximum element size was 5×10^−8^ m, minimum element size 1×10^−9^ m, maximum element growth rate 1.1 and resolution of curvatures 0.2. All the boundaries in the protrusion including the gap junction area had resolution maximum of 5×10^−9^ m, minimum 5×10^−10^ m and a maximum growth rate of 1.2. When the radius of the gap junction in Model 2 was reduced the maximum element size was 1×10^−9^ m around the boundary defined by the gap junction. The maximum growth rate defines how much the element size can grow from a region with smaller elements to a region with larger elements. A maximum growth rate of 1.2 means that the element size can increase by 20% from one element to the next.

### Model 1

In Model 1 we simulated diffusion of Ca^2+^ ions in a non-buffered cytosol in order to quantify the basic properties of the structure. Initially the concentration of Ca^2+^ was 0.1 µM in all compartments. We simulated an increase in bulk cytoplasmic Ca^2+^ concentration in either the EC or SMC by increasing the boundary concentrations (Fig. 1C, green lines) by 0.5 µM to a final concentration of 0.6 µM. Unless explicitly stated the diffusion of Ca^2+^ was assumed to be isotropic with a diffusion coefficient of 233 µm^2^/s [18].

All parameters are listed in Table 2 and initial conditions in Table 3.

**Table 2:** List of parameters in the models.

Parameter	Value	Unit	Description	See Reference
*k_on_*	50×10^6^	1/((mol/l) s)	Rate constant, Ca^2+^ buffer cytosolic	[32]
*k_off_*	25	1/s	Rate constant, Ca^2+^ buffer cytosolic	[32]
*k_on,ER_*	10×10^4^	1/((mol/l) s)	Rate constant, Ca^2+^ buffer ER	[21]
*k_off,ER_*	200	1/s	Rate constant, Ca^2+^ buffer ER	[21]
*D_Ca_*	233	µm^2^/s	Diffusion coefficient of Ca^2+^	[18]
*D_buffer_*	13	µm^2^/s	Diffusion coefficient of Ca^2+^ buffer	[18]
*D_bound_*	13	µm^2^/s	Diffusion coefficient of Ca^2+^ buffer bound to Ca^2+^	[18]
*D_IP3_*	280	µm^2^/s	Diffusion coefficient of IP_3_	[18]
a_4_	0.2×10^6^	1/((mol/l) s)	Rate constant for IP_3_R	[22]
a_5_	20×10^6^	1/((mol/l) s)	Rate constant for IP_3_R	[22]
d_1_	0.13×10^−6^	mol/l	Equilibrium constant for IP_3_R	[22]
d_2_	1.049×10^−6^	mol/l	Equilibrium constant for IP_3_R	[22]
d_3_	943.4×10^−9^	mol/l	Equilibrium constant for IP_3_R	[22]
d_4_	144.5×10^−9^	mol/l	Equilibrium constant for IP_3_R	[22]
d_5_	82.34×10^−9^	mol/l	Equilibrium constant for IP_3_R	[22]
*V_SERCA_f_* and *V_SERCA_r_*	1.022×10^−7^	mol/(m^2^ s)	SERCA pump, max capacity	[21]
*K_SERCA_f_*	260×10^−9^	mol/l	SERCA pump, affinity	[21]
*H_SERCA_f_* and *H_SERCA_r_*	0.75	-	SERCA pump, cooperativity	[21]
*K_SERCA_r_*	1.8×10^−3^	mol/l	SERCA pump, affinity	[21]

**Table 3:** List of variables and initial conditions.

**Variable**	**Initial value**	**Unit**	**Description**
Ca^2+^ (cytosol)	0.1	µM	Ca^2+^ concentration in the cytosol
Ca^2+^ (ER)	500	µM	Ca^2+^ concentration in ER
IP_3_	0.1	µM	IP_3_ concentration
Buffer (cytosol)	83.3	µM	Ca^2+^ buffer (free form) in the cytosol
Buffer (ER)	57.6	mM	Ca^2+^ buffer (free form) in ER
Ca^2+^, Bound (cytosol)	16.7	µM	Ca^2+^ buffer (bound to Ca^2+^) in the cytosol
Ca^2+^,Bound (ER)	14.4	mM	Ca^2+^ buffer (bound to Ca^2+^) in ER
x_000_	0.265	1	Fraction of IP_3_R on free form
x_001_	0.185	1	Fraction of IP_3_R with Ca^2+^ bound to the activating site
x_010_	0.324	1	Fraction of IP_3_R with Ca^2+^ bound to the inhibitory site

### Model 2

The model was extended to include the effect of Ca^2+^-induced Ca^2+^-release (CICR) from *endoplasmic reticulum* (ER) in the EC and MEJ. This model contains the following diffusible species: Ca^2+^ in the cytosol and in ER, Ca^2+^ buffers in the cytosol and ER and IP_3_ in the cytosol (see Table 2 for diffusion coefficients). We assumed that all pumps and channels were distributed uniformly in the membranes of the ER and hence any effects from point sources were neglected. The structure of the ER inside the MEJ was based on electron microscopic images [7]. The ER in the EC had a radius of 6 µm and was 0.5 µm thick and 0.1 µm from the upper part of the EC membrane. In the inclined part of the MEJ neck the latter distance was 60 nm and in the vertical part it was 75 nm. In the head of the MEJ the ER had a radius of 0.25 µm (Fig. 1D–E).

We also included the reversible binding of Ca^2+^ to Ca^2+^ buffers in the cytosol of the EC, MEJ and SMC,
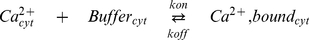
(2)with the two rate constants *k_on_* and *k_off_* (see Table 2). The reaction was modeled using first and second order kinetics.



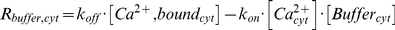
(3)The ratio between *k_off_* and *k_on_* gives the dissociation constant Kd (Kd = *k_off_*/*k_on_*), which corresponds numerically to the Ca^2+^ concentration at which the buffer is 50% saturated. In the cytosol of the SMC, EC and MEJ the Kd was 0.5 µM [19], [20]. The total buffer concentration was 0.1 mM. Ca^2+^ buffer binding was also modeled in ER:
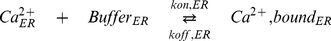
(4)which was modeled as in the cytosol, see Eq.
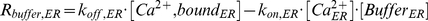
(5)where the total Ca2+ buffer capacity was 72 mM, with a Kd value of 2 mM [21]. We modeled CICR using the model by De Young and Keizer [22] which we adapted to the present model. The flux of Ca2+ through the IP3 receptor channel (IP3R) was dependent on the permeability of the channel (PIP3R), the fraction of channels being in an open state 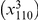 and the Ca2+ gradient across the ER membrane. The flux across the ER membrane was then:



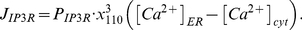
(6)The IP_3_ receptor is activated by IP_3_ and by low concentrations of Ca^2+^, whereas Ca^2+^ at higher concentrations inhibits the channel. Using a rapid equilibrium approximation De Young and Keizer [22] presented a set of equations to describe the different states of the receptor. Each position in the subscript (x_ijk_) describes if a ligand is bound (1) or not bound (0) to the three independent binding sites. The first place (i) refers to binding of IP_3_, the second (j) refers to binding of Ca^2+^ to the activating site and the third (k) to binding of Ca^2+^ to the inhibitory site.

In the model it is assumed that the active channel is composed of three subunits and because it is assumed that all three have to be in the state x_110_ for the channel to be open, the fraction of open channels is given by 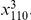 The dynamical change between the forms without IP_3_ is described with the following equations [22];.
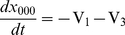
(7)

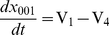
(8)

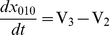
(9)

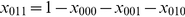
(10)where 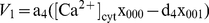
(11)

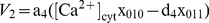
(12)

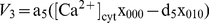
(13)

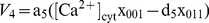
(14)and the IP_3_ bound forms are found as:
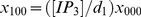
(15)

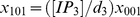
(16)

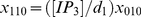
(17)

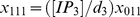
(18)where a_x_ and d_x_ are constants (Table 2).

Re-uptake of Ca^2+^ into the ER by the SERCA pump (a Ca^2+^ pump) was modeled using [21].
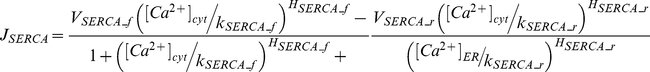
(19)where the *f* and *r* indicates the forward and reverse modes. All constants are given in Table 2 and initial conditions in Table 3.

The IP_3_ concentration was 0.1 µM prior to stimulation and the maximum Ca^2+^ permeability through the IP_3_R channel (*P_IP3R_*) was fitted to counterbalance the activity of the SERCA pump [21]. To simulate an increase in IP_3_ in the EC or the SMC we increased the concentration of IP_3_ to 0.4 µM around all external boundaries in either the EC or the SMC (Fig. 1C). Note that the diffusion coefficient of IP_3_ is higher than that of Ca^2+^ (Table 2).

## Results

**Figure 2:**
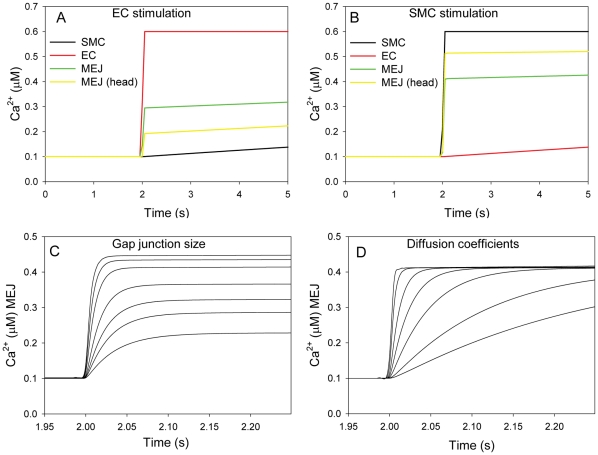
Model 1, diffusion through the MEJ. The effect of the MEJ on diffusion between the SMC and EC was tested by increasing the Ca^2+^ concentration in A): EC from 0.1 µM to 0.6 µM and B): in the SMC from 0.1 µM to 0.6 µM at time 2 s. The average concentration in the EC, the SMC, the MEJ and only in the head of the MEJ is shown in both cases. C) Average Ca^2+^ concentrations in the MEJ as a function of gap junction pore size. From below pore size was: 10, 20, 30, 50, 100, 150 and 200 nm. D) The diffusion coefficient was changed within a chemical/biological realistic regime and the average Ca^2+^ concentration in the MEJ was found for each value. From below 10, 20, 50, 100, 200, 500 and 1000 µm^2^/s.

To elucidate the effect of the MEJ on diffusion between the two cell types, we considered a simple system of Ca^2+^ diffusion in an unbuffered system and we evaluated two scenarios where either the EC or the SMC was stimulated by increasing the bulk cytosolic Ca^2+^ concentration (Model 1). An increase in bulk Ca^2+^ concentration was simulated by raising the concentration of cytosolic Ca^2+^ ([Ca^2+^]_cyt_) by 0.5 µM above the resting level at all boundaries in either of the cells, except for the boundaries of the MEJ (see Fig. 1 for details), and we assumed initially that the cytosol was unbuffered. Following perturbation of the Ca^2+^ concentration, the bulk cytosolic concentration increased to almost 0.6 µM within 10 ms in the EC and within 30 ms in the SMC (Fig. 2A and B). The slower increase in the SMC is due to the lower surface to volume ratio. The fast increase in concentration shows that raising the concentration at the boundaries was a good approximation of simulating a global perturbation in the cell.

While the Ca^2+^ response in the stimulated cell was very fast, the bulk cytosolic Ca^2+^ concentration in the other cell responded very slowly with only small changes in bulk cytosolic Ca^2+^ levels even after several seconds (Fig. 2A and B). Whereas the responses in the bulk cytosolic Ca^2+^ concentrations were quite similar in the two cases, the changes in the Ca^2+^ concentration in the microdomain formed by the MEJ and the head of the MEJ differed substantially. It is noteworthy that whereas the Ca^2+^ signal that originated from the EC only had a modest effect on the head and the neck of the MEJ (Fig. 2A), the Ca^2+^ signal from the SMC increased the Ca^2+^ level in the head of MEJ, which is a part of the EC, to >0.5 µM within 40 ms (Fig. 2B). This shows that a signal from the cell that makes contact with the MEJ, here the SMC, can be transmitted very rapidly to the other cell, but less easily in the opposite direction. The rectification was caused by the direct coupling of the MEJ head to the SMC. Because of the geometry Ca^2+^ diffuses easily from the SMC to the MEJ head. Since the bulk of the EC is separated from the MEJ by a narrow neck the efflux from the MEJ head into the bulk of the EC is delayed. The SMC can therefore easily drive an increase in MEJ Ca^2+^ concentration. In contrast, a concentration increase in the bulk cytoplasm of the EC will only lead to a slow increase in the concentration in the head of the MEJ. In addition compounds that diffuse from the EC into the MEJ head will easily diffuse into the SMC, and the effect on the concentration in the head will be minimized.

The permeability of the gap junction and the diffusion coefficient could be central features of the model. We therefore tested how the increase in MEJ Ca^2+^ concentration in response to an increase in the SMC Ca^2+^ concentration, varied with changes in gap junction size (i.e. the degree of coupling) (Fig. 2C) and diffusion coefficient (Fig. 2D). We found that the steady-state concentration in the MEJ decreased when the gap junction size was lowered, whereas the rise time was almost unaffected. In contrast, a general decrease in the diffusion coefficient prolonged the rise time for the increase in MEJ concentration, but the steady state concentration was unaffected (Fig. 2D).

**Figure 3:**
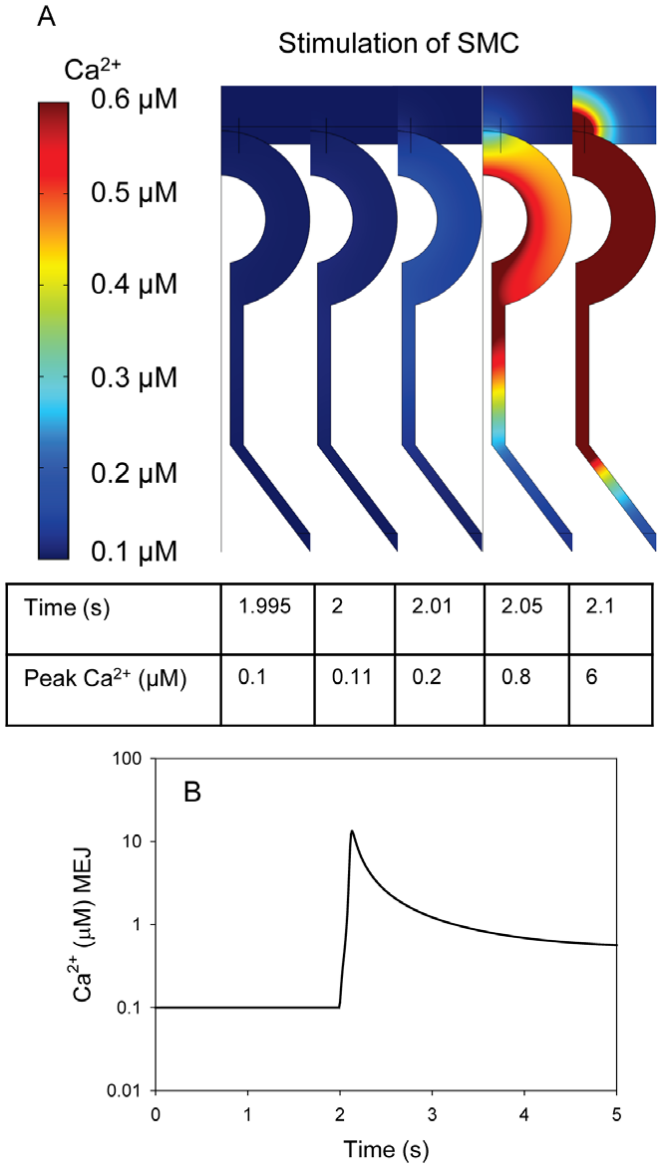
Model 2, the MEJ is a sensor of the adjacent cell. A) The changes in Ca^2+^ concentration in the protrusions following an increase in the boundary level of IP_3_ in the SMC from 0.1 to 0.4 µM. The local Ca^2+^ cloud in the SMC is due to diffusion of Ca^2+^ from the EC/MEJ through the gap junction. The concentration of Ca^2+^ in the protrusion is shown at the indicated time points. Below the bar with the time points are the peak concentrations of Ca^2+^. The IP_3_ level was increased at time 2 s in the SMC. B) Average Ca^2+^ concentration in the MEJ following an IP_3_ increase in the SMC. When IP_3_ was increased from 0.1 to 0.4 µM in the SMC the Ca^2+^ microdomain in the MEJ was on average of 4 µM at time 2.1 s and 12 µM at time 2.14 s.

**Figure 4:**
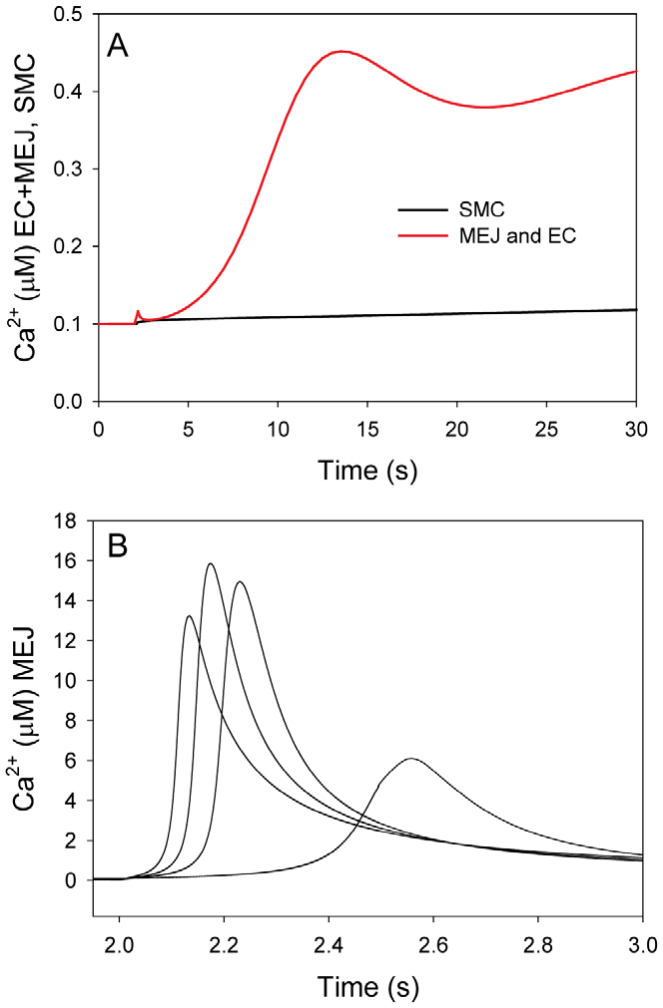
The MEJ amplifies signals from the SMC to the EC. Following an increase in SMC IP_3_ concentration from 0.1 to 0.4 µM, Ca^2+^ increased in the MEJ. A) When IP_3_ was increased to 0.4 µM in the SMC the global bulk EC [Ca^2+^]_cyt_ increased to >0.3 µM within 5–10 s in the EC, but the [Ca^2+^]_cyt_ in the SMC remained unaffected. B) The radius of the pore was set to the following values (curves from the right): 10, 30, 50 and 100 nm, and average MEJ concentration was calculated following an increase in IP_3_ in the SMC.

We next considered the presence of an amplifier in the MEJ by including a model of the *endoplasmic reticulum* (ER) (Model 2). ER is present in the MEJ and it may extend into the cytosol of the EC [6], [7]. Although it is unknown how often this is the case, we assumed in the model that the ER in the MEJ couples to the ER in the cytosol (Fig. 1D and E). The ER surface, including that inside the MEJ, contains IP_3_ receptor channels (IP_3_R) [6], [7] enabling Ca^2+^ induced Ca^2+^ release (CICR) [23]. When IP_3_ was increased in the SMC there was a pronounced Ca^2+^ release in the MEJ after less than 100 ms due to Ca^2+^ release from the ER (Fig. 3). MEJ Ca^2+^ concentration increased rapidly (<50 ms) after SMC IP_3_ was an increased (Fig. 3A) and the average Ca^2+^ concentration in the MEJ remained high for more than 1 s (Fig. 3B). When an IP_3_ increase was generated in the SMC, Ca^2+^ from the ER in the EC will elevate the EC [Ca^2+^]_cyt_, (Fig. 4A), but the reverse flux of Ca^2+^ from the EC to the SMC will only have a marginal effect on SMC [Ca^2+^]_cyt_ (Fig. 4A).

The permeability of the gap junctions in the MEJ is to the best of our knowledge not known, and to address the impact of that we tested the model for various sizes of the gap junction pore (Fig. 2C and Fig. 4B). We found that over a range of gap junction sizes from 10–200 nm an increase in IP_3_ in the SMC was able to generate a substantial Ca^2+^ signal in the MEJ as seen by the increase in MEJ Ca^2+^ concentration (Fig. 4B). The simulation when the pore was 200 nm was similar to the simulation where the pore was 100 nm (not shown).

**Figure 5:**
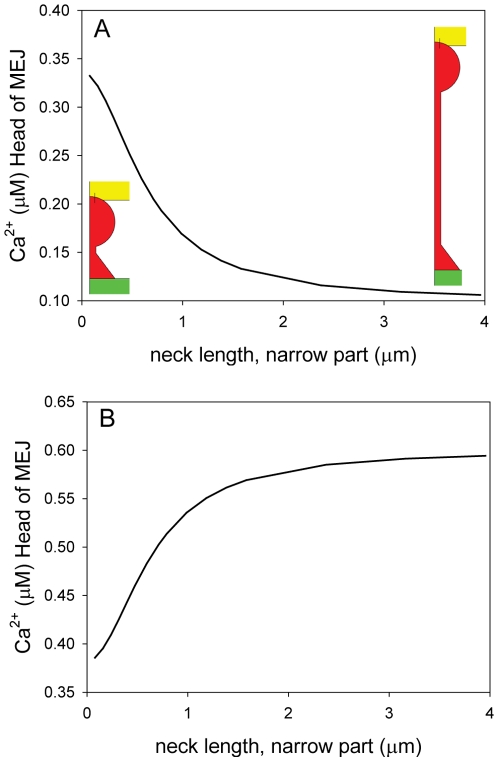
The length of the MEJ. The effect of changing the length of the narrow/vertical part of the neck of the myoendothelial junction was tested on the model only including Ca^2+^ (Model 1). Re-scaling the length of the neck was implemented by re-scaling the diffusion coefficient in the z-axis in the neck. The value used in Fig. 1, 2, 3, and 4 was 1.26 µm. To quantify the effect of neck re-scaling, the average bulk Ca^2+^ concentration in the head of the myoendothelial junction was measured 0.1 s after stimulation when A): the EC was stimulated and B): when the SMC was stimulated.

We addressed the possibility of variation in the length of the MEJ by modeling changes in the length of the longitudinal part of the neck that connects the head with the EC body. For this purpose we used Model 1 that only includes one compound (Ca^2+^). To model variations in the length of the neck the diffusion coefficient was re-scaled in the direction of the z-axis in the narrow part of the neck (Fig. 5). Changes in length will scale with the diffusion coefficient multiplied by the inverse of the scaling factor raised to the power of two, i.e. quadrupling the diffusion coefficient corresponds to halving the length. To quantify the effect of changing the length of the neck we simulated the average Ca^2+^ concentration in the head of the MEJ 0.1 s after stimulation of one of the cell types. When the EC was stimulated a decreased length of the neck increased the Ca^2+^ concentration in the head of the MEJ after stimulation whereas a longer neck decreased the concentration (Fig. 5A). When the SMC was stimulated a longer neck increased the Ca^2+^ concentration in the head of the MEJ (Fig. 5B). Hence, a longer neck will increase the polarization effect. If the MEJ was removed from the model and the EC coupled directly to the SMC, we only observed a minor change of 0.018 µM after 0.1 s in the cell opposite of the one that was activated (not shown). Hence, without the local environment provided by the MEJ a signal from the SMC is diluted in the EC. We have also tested the effect of re-scaling the neck in the model including CICR (Model 2, not shown) and we found that increasing the length of the neck with a factor of 2–3 still allowed an IP_3_ signal from the SMC to initiate a global Ca^2+^ signal in the EC within a few seconds. The models predict that for different lengths of the neck the structure can rectify a signal between the two cells and increasing the length of the neck will increase that effect.

## Discussion

ECs and SMCs are often connected by gap junctions that are located on MEJs. The distribution of ion channels near the MEJ appears to be tightly regulated and there is evidence that the local environment i.e. ion channel localization is highly controlled [24]. We have investigated the impact of the structure of the MEJ using a 2D axi-symmetric mathematical model and we found that the structure of the MEJ itself can influence the signaling between the two cell types by rectifying information flow.

We perturbed the model by simulating an increase in the bulk cytosolic concentration of a diffusible species in either the EC or the SMC. The increase in concentration was implemented as a concentration increase at the boundaries in the respective cells (Fig. 1C). The lag time between bulk cytosolic concentration had reached the level at the boundary from the time it was changed was only 10–30 ms. Since we expect signals originating from the lumen of the vessel to primarily act on the part of the EC membrane facing the lumen, and to a much lesser extent affect the cell membrane in the sequestered region of the MEJ, we deliberately excluded the MEJ from the rise in the cytosolic Ca^2+^ concentration. The main conclusion from the results shown in Figure 2 is that for a small diffusible species (in this case Ca^2+^) that can pass through gap junctions, the structure of the MEJ leads to significant signaling rectification in the sense that the concentration of the diffusible species in the head responds more rapidly to changes in the SMC than it does to changes in the EC from which it originates.

The degree of asymmetry depends on two factors, the degree of gap junctional coupling between the head and the SMC and the length of the neck region that connects the head to the bulk of the EC. As can be seen in Figure 2C, increasing the degree of coupling increases the steady state concentration achieved in the head, but only has a minor effect on the time it takes to reach this level. This is to be expected, since in the model, the steady state concentration will be determined by a balance between the influx from the SMC and the loss of through the neck region. The importance of the length of the neck region is illustrated in Figures 5A and B where we simulated various lengths of the neck region by varying the magnitude of the diffusion coefficient in the direction of the long axis of the neck region. Imposing anisotropic diffusion conditions is equivalent to changing the length of the neck region. A longer neck region will impede the diffusion of solutes from the bulk cytosol of the EC into the head of the MEJ. This effect can be seen in Figure 5A which shows the Ca^2+^ concentration in the head 0.1 s after stimulation of the EC. It is clear that the longer the neck, the smaller the concentration obtained. The reverse was true for the response of the head to an increase in SMC Ca^2+^ concentration, where a longer neck hindered the loss of Ca^2+^ from the head, and therefore led to a higher concentration following the stimulation (Fig. 5B). The results clearly indicate that the micro-anatomy of biological systems may have an important role in signal transduction and modulation.

We next addressed the question of how a change in head concentration of a given substance may affect the EC. One possibility could be the presence of an amplification mechanism within the head region. It is well known that the ER may extend into the MEJ, and we therefore considered the possible role of the ER in signal amplification. In this scenario we modeled the diffusion of IP_3_, and as in the former case, the head region quickly equilibrated with respect to changes in IP_3_ levels in the SMC. Because of the presence of the ER, the rise in the IP_3_ level in the head will lead to Ca^2+^ release from the ER through the IP_3_ receptors. Such a localized Ca^2+^ release may then spread into the EC as a Ca^2+^ wave mediated by Ca^2+^ induced Ca^2+^ release (Fig. 3). We hypothesize that such an amplification mechanism may effectively propagate signals from the SMC into the EC. Indeed, recent experiments have suggested that IP_3_ from the SMC can diffuse through the gap junctions in the MEJs and induce a local Ca^2+^ increase in the EC [25], [26].

The restricted space in the MEJ is an example of a local Ca^2+^ microdomain, i.e. a restricted volume of the cytoplasm where the local Ca^2+^ concentration may reach high levels (Fig. 2 and 3). Another example of a Ca^2+^ microdomain is the restricted space between endoplasmic/sarcoplasmic reticulum and the plasma membrane [27]–[29], close to ion channels [30], [31]. The wrinkled structures of the plasma membrane in neutrophil granulocytes have also been suggested to give rise to Ca^2+^ microdomains where the local concentration of Ca^2+^ can reach values of more than 10 µM [32], [33].

Ca^2+^ microdomains have important roles in regulation of processes near membranes including regulation of ion channels [27]–[31]. In small arteries Ca^2+^ microdomains in the SMCs regulate the activity of BK channels and thereby play a central role in regulation of the tone [29], [34]. In the ECs from small arteries the spatial distribution of K^+^ channels is tightly regulated [24]. The K^+^ channel type KCa3.1 is highly expressed in the MEJ [8], [24], [35] and KCa2.3 is present in the MEJ and at the cell border [24]. KCa3.1 and KCa2.3 are both activated by Ca^2+^ in the range 50–900 nM [36], and hence an increase in MEJ Ca^2+^ concentration could activate K^+^ currents in the MEJ, which in turn would hyperpolarize the entire cell. Activation of the channel would in addition lead to an increase in the K^+^ concentration in the internal elastic lamina, which could, in principle, activate Kir channels in both EC and SMCs. It is known that an increase in interstitial K^+^ concentration dilates vessels in part due to Kir activation [37]–[40]. It is therefore plausible that activation of K^+^ channels in the MEJ could lead to a hyperpolarization of the SMC, in turn inhibiting voltage sensitive Ca^2+^ channels leading to vasodilation.

It has previously been suggested that asymmetrical distribution of IP_3_Rs and IP_3_ phosphatases also can give rise to an asymmetrical IP_3_/Ca^2+^ signaling between ECs and SMCs [14], [41], and we speculate that these effects could act together with the structural effects presented here. Knowledge of the distribution of IP_3_Rs in and near MEJ in arterioles from various tissues is limited. However, our conclusions are not dependent on any functional polarization, but only depend on the actual structure of the MEJ. If degradation of IP_3_ by phosphatases is taken into account it will enhance the reported effect of a unidirectional flow from the SMC to the EC, because IP_3_ that enters the MEJ from the SMC has a relative short distance to travel before it encounters the ER in the MEJ. IP_3_ produced in the EC will on the other hand have to travel through the entire MEJ before entering the SMC and this would decrease the amount of IP_3_ entering the SMC. In the SMC IP_3_ could activate opening of IP_3_Rs in the sarcoplasmic reticulum, but as we have shown in Figures 2 and 3 the flow of molecules from the EC to the SMC is operating on a time scale that is substantially slower than the flow from the SMC to the EC. Hence, under normal conditions it would be unlikely that IP_3_ produced in the EC could activate IP_3_R channels in the SMC. If phosphatases in both ECs and SMCs were inhibited and the EC was stimulated for a prolonged period it could be speculated that IP_3_ from the EC could stimulate a Ca^2+^ release in the SMC.

In conclusion, the micro-anatomical structure of the MEJ, with a long neck region restricting diffusion between the head and the bulk of the cytosol, by itself leads to a rectification of information flow between the SMC and the EC. Changes in IP_3_ and Ca^2+^ in the SMC are rapidly and efficiently transmitted to the EC, whereas the reverse is not the case.
